# Cell passage number drives transcriptomic drift as an overlooked factor in experimental reproducibility

**DOI:** 10.1038/s41598-025-29424-1

**Published:** 2025-11-21

**Authors:** Shuai Liu, Hailun Peng, Junyan Meng, Liyuan Zhuo, Shaoping Fu, Wei Yang

**Affiliations:** 1 Department of Laboratory, The Fourth People’s Hospital of Shenzhen (Shenzhen Samii Medical Center), Shenzhen, 518118 Guangdong China; 2 Department of Critical Care Medicine, Longgang Central Hospital (Shenzhen Clinical College, Guangzhou University of Chinese Medicine), Shenzhen 6082 Longgang Road, Shenzhen, 518116 Guangdong China

**Keywords:** Cell passage, Reproducibility, Cell culture, Cancer, Computational biology and bioinformatics

## Abstract

The reproducibility of scientific research has been increasingly challenged in recent years. While extrinsic factors (e.g., cross-contamination, mycoplasma infection, serum variability) are well-studied, the role of intrinsic attributes like cell passage number remains underexplored. Using two tumor cell lines (ACHN and Renca), we employed RNA sequencing to analyze transcriptomic dynamics across passages (P3 to P39). Results revealed a nonlinear transcriptomic shift: mid-passage cells (P10/P11, P17) showed heightened activity in cell cycle, metabolism, and stress response, whereas low (P3) and high passages (P24/P39) exhibited stable and similar profiles. KEGG analysis indicated significant alterations in signaling, immune, and metabolic pathways during passaging. This study demonstrates that passage number shapes transcriptional landscapes and impacts experimental reproducibility. We advocate for strict passage control and explicit reporting of passage information to enhance reliability. These findings underscore the need for standardized cell culture practices to improve research reproducibility.

**Supplementary Information:**

The online version contains supplementary material available at 10.1038/s41598-025-29424-1.

## Introduction

Recent years have witnessed rapid technological advancement, leading to an unprecedented generation of scientific data that has greatly accelerated research progress and the exploration of biological mechanisms. However, a survey conducted by Baker in 2016 revealed that nearly 80% of biological researchers were unable to reproduce others’ experimental results, with 60% failing to replicate their own experiments^1^. This phenomenon has cast a shadow over the credibility and sustainable development of scientific research.

Animal cell culture technology, which maintains cell growth, proliferation, and basic structure and function by simulating the in vivo environment in vitro, has become a widely used core methodology in cell biology research. To date, cell-based models have accumulated vast amounts of experimental data covering various fields including gene expression regulation, protein interaction networks, drug screening, and disease mechanisms, fully demonstrating the important role of cell platforms in modern life science research. However, significant discrepancies often exist in research results obtained by different laboratories using the same cell line, making the reproducibility of experimental results an increasingly prominent issue. This problem not only affects the reliability of scientific conclusions but also leads to substantial waste of human, material, and financial resources.

Existing studies have shown that extrinsic factors such as cell line misidentification, cross-contamination, poor annotation^2,3^, mycoplasma infection^4–7^, and fetal bovine serum variations^8,9^ are among the main causes of irreproducibility in cell experiments. However, compared to these extensively studied extrinsic factors, the impact of intrinsic cellular attributes on experimental results remains systematically underinvestigated, particularly regarding the critical variable of cell passage number. This factor may influence experimental reproducibility by affecting cellular gene expression. Therefore, conducting in-depth and systematic evaluation of the impact of cellular intrinsic factors on experimental reproducibility is of great significance for enhancing the scientific rigor and reliability of research.

In this study, we hypothesize that cell passage number serves as a critical intrinsic factor influencing the reproducibility of cell-based experiments by inducing dynamic transcriptomic alterations during serial passaging. To test this hypothesis, we selected two commonly used tumor cell lines, Renca and ACHN, as research models. After thawing (designated as P0), cells were continuously passaged at a 1:4 ratio up to P39. Cells were collected at passages P3, P10/11, P17, P24, and P39 for RNA sequencing analysis. This comprehensive transcriptome-level assessment aims to (1) reveal gene expression dynamics across passages, (2) elucidate the intrinsic biological changes during passaging, and (3) to provide a theoretical basis and critical awareness for understanding the potential mechanisms by which cell passaging affects experimental reproducibility.

## Materials and methods

### Cell culture

ACHN and Renca cell lines were purchased from Procell Life Science & Technology Co., Ltd. (originating from ATCC) and authenticated by STR profiling. We cultured cells in DMEM (#C11995500BT, Gibco) with 10% FBS (#FSP500, ExCell Bio) at 37 °C in a humidified incubator with 5% CO₂. Following thawing (designated P0), cells were passaged at a 1:4 ratio every 3 days upon reaching 80–90% confluence. To minimize batch-to-batch variation, a single large batch of DMEM and FBS was used throughout the entire passaging process from P0 to P39. We tested for mycoplasma contamination (Vazyme) before every passage and at the experiment endpoint, and all results were negative.

### RNA sequencing

Total RNA was extracted using Trizol reagent. RNA purity was assessed with a NanoPhotometer® spectrophotometer (IMPLEN, CA, USA), concentration was measured using a Qubit® 2.0 Fluorometer (Life Technologies, CA, USA), and integrity (RIN value) was evaluated with an Agilent 2100 Bioanalyzer (Agilent Technologies, CA, USA). After quality control, mRNA was enriched using Oligo(dT) magnetic beads, and first-strand cDNA was synthesized with M-MuLV Reverse Transcriptase. RNA templates were degraded by RNase H, and second-strand cDNA was synthesized. Subsequent steps included end repair, poly-A tailing, adapter ligation, and size selection (~ 200 bp) using AMPure XP beads, followed by PCR amplification for library construction.

Libraries passing quality control were subjected to paired-end sequencing on an Illumina platform. Raw reads were processed with fastp (v0.23.4) to remove low-quality reads, adapter contaminants, and reads with excessive N content, yielding clean reads. rRNA contamination was eliminated by aligning reads to an rRNA database using bowtie2 (v2.4.4). The remaining reads were mapped to the reference genome using HISAT2 (v2.2.1), and mapping efficiency was calculated. Gene expression levels (FPKM) were estimated with StringTie (v1.3.4d) and RSEM (v1.3.3). Differential expression analysis was performed using DESeq2 (v1.24.0) with thresholds of FDR < 0.05 and |log_2_Fold Change|> 1.

Based on differentially expressed genes, principal component analysis (PCA), sample correlation analysis, and cluster heatmaps were generated using R (v 3.14.0). Gene Ontology (GO) annotation covered biological process (BP), cellular component (CC), and molecular function (MF) categories. Pathway enrichment analysis was conducted based on the KEGG database, with statistical significance evaluated by adjust *p* value < 0.05.

## Results

### Transcriptomic profiling of cells at different passage numbers

**Fig. 1:**
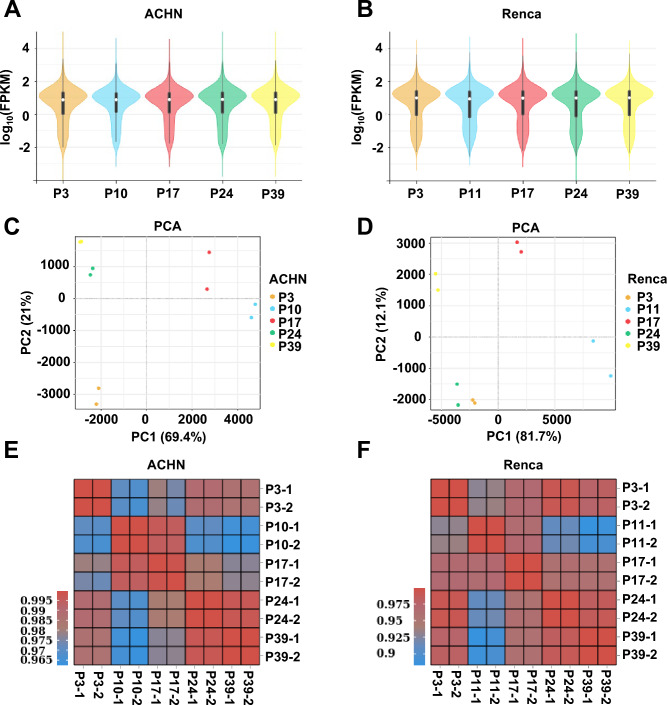
RNA-seq expression profiling of ACHN and Renca cell lines at different passage numbers. (**A**, **B**) Violin plots showing the distribution of gene expression levels in ACHN (**A**) and Renca (**B**) cells at different passages (P3, P10/P11, P17, P24, P39). Each group demonstrates consistent intra-sample expression distribution. (**C**, **D**) Principal component analysis (PCA) illustrating the overall trajectory of transcriptomic changes in ACHN (**C**) and Renca (**D**) cells across passages. Each point represents an individual sample, with colors indicating different passage time points. (**E**, **F**) Pearson correlation heatmaps depicting gene expression correlations among all samples for ACHN (**E**) and Renca (**F**) cells.

To investigate the impact of cell passage number on intrinsic gene expression and its potential influence on experimental outcomes, we continuously cultured ACHN and Renca cell lines and selected cells at low (P3), intermediate (P10/11, P17), and high (P24, P39) passages for RNA-seq analysis. Violin plots revealed a similar overall distribution of expression levels across all time points, indicating consistent sequencing data quality (Fig. 1A, 1B). Principal component analysis (PCA) demonstrated that cells gradually dispersed in the principal component space with increasing passages, suggesting significant alterations in gene expression profiles over serial passaging (Fig. 1C, 1D). Furthermore, sample correlation heatmaps confirmed high reproducibility among biological replicates at the same time point, while revealing progressive transcriptomic divergence across different passages, supporting systematic remodeling of the transcriptome during long-term culture (Fig. 1E, F).

### Analysis of differentially expressed genes across passages

**Fig. 2:**
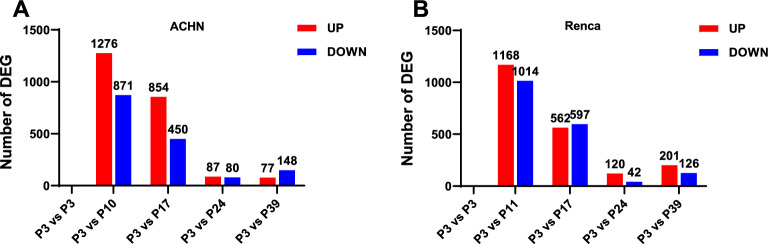
Quantitative changes of DEGs in ACHN and Renca cells at different passages compared to P3. Number of DEGs in ACHN cells at P10, P17, P24, and P39 passages relative to P3. Number of DEGs in Renca cells at P11, P17, P24, and P39 passages relative to P3. Red bars represent up-regulated genes (UP), blue bars indicate down-regulated genes (DOWN), with numerical values showing the quantitative changes of DEGs.

To further characterize the extent of transcriptomic changes at different passage stages, we compared the number of differentially expressed genes (DEGs) at each subsequent time point using early-passage cells (P3) as the reference. In ACHN cells, 1,276 up-regulated and 871 down-regulated DEGs were identified at P10, while 854 up-regulated and 450 down-regulated DEGs were detected at P17. In contrast, significantly fewer DEGs were observed at higher passages (P24 and P39) (Fig. 2A, Supplementary Material 1). Renca cells exhibited a similar trend: 1,168 up-regulated and 1,014 down-regulated DEGs were identified at P11, declining to 562 up-regulated and 597 down-regulated DEGs at P17. By P39, only 201 up-regulated and 126 down-regulated DEGs were detected (Fig. 2B, , Supplementary Material 1).

These results indicate a nonlinear pattern of gene expression changes during serial passaging in both cell lines, characterized by significant intermediate fluctuations and relative stability at both ends This suggests a dynamic transcriptomic remodeling process rather than a simple linear trend.

### Dynamic changes of gene expression profiles in ACHN and Renca cells across passages

**Fig. 3:**
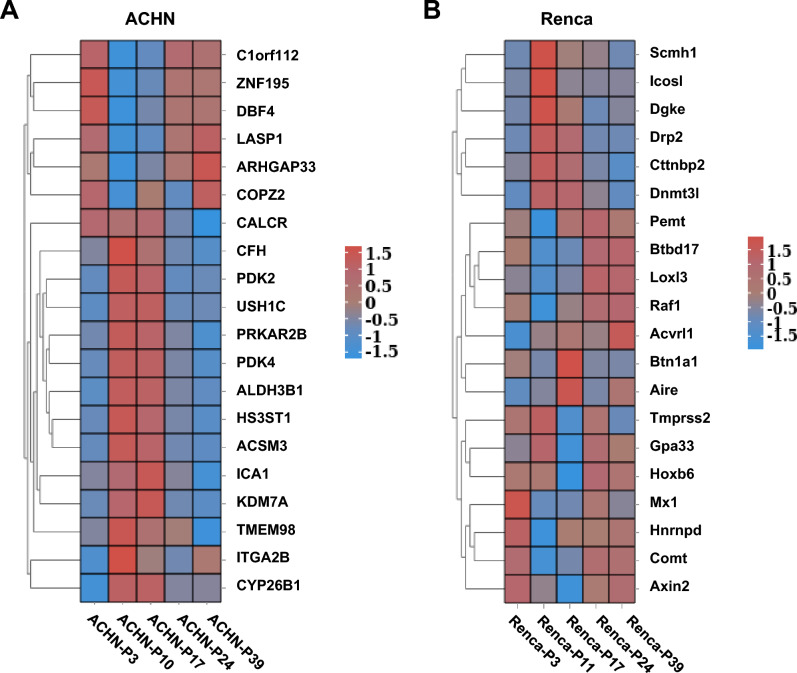
Cluster analysis of DEGs across passages. Heatmap of representative DEGs in ACHN cells (n = 2) at P3, P10, P17, P24, and P39 passages. Heatmap of representative DEGs in Renca cells (n = 2) at P3, P11, P17, P24, and P39 passages. Shown is a heatmap of Z-scores (based on log_10_(FPKM + 1) values) for representative DEGs. Genes were clustered by Euclidean distance. Red and blue colors indicate up- and down-regulation, respectively.

To further elucidate the expression changes of key genes during continuous passaging, we performed cluster heatmap analysis of the identified DEGs. In ACHN cells, multiple genes exhibited significant up- or down-regulation patterns across different passages, suggesting their potential involvement in critical biological processes such as metabolic regulation (e.g., PDK2, PDK4) and cell cycle control (e.g., DBF4) during passaging (Fig. 3A). Similarly, Renca cells showed dynamic expression changes of multiple genes across passages, including Btbd17, Loxl3, Raf1, and Acvrl1, which may be closely associated with signal transduction, transcriptional regulation, and cancer-related pathways (Fig. 3B). Collectively, both cell lines exhibited systematic remodeling of gene expression patterns with increasing passages, reflecting active or passive adaptation of gene expression during long-term in vitro culture. These findings may have important implications for the stability and reproducibility of experimental results.

### Functional enrichment analysis of DEGs during passaging

**Fig. 4:**
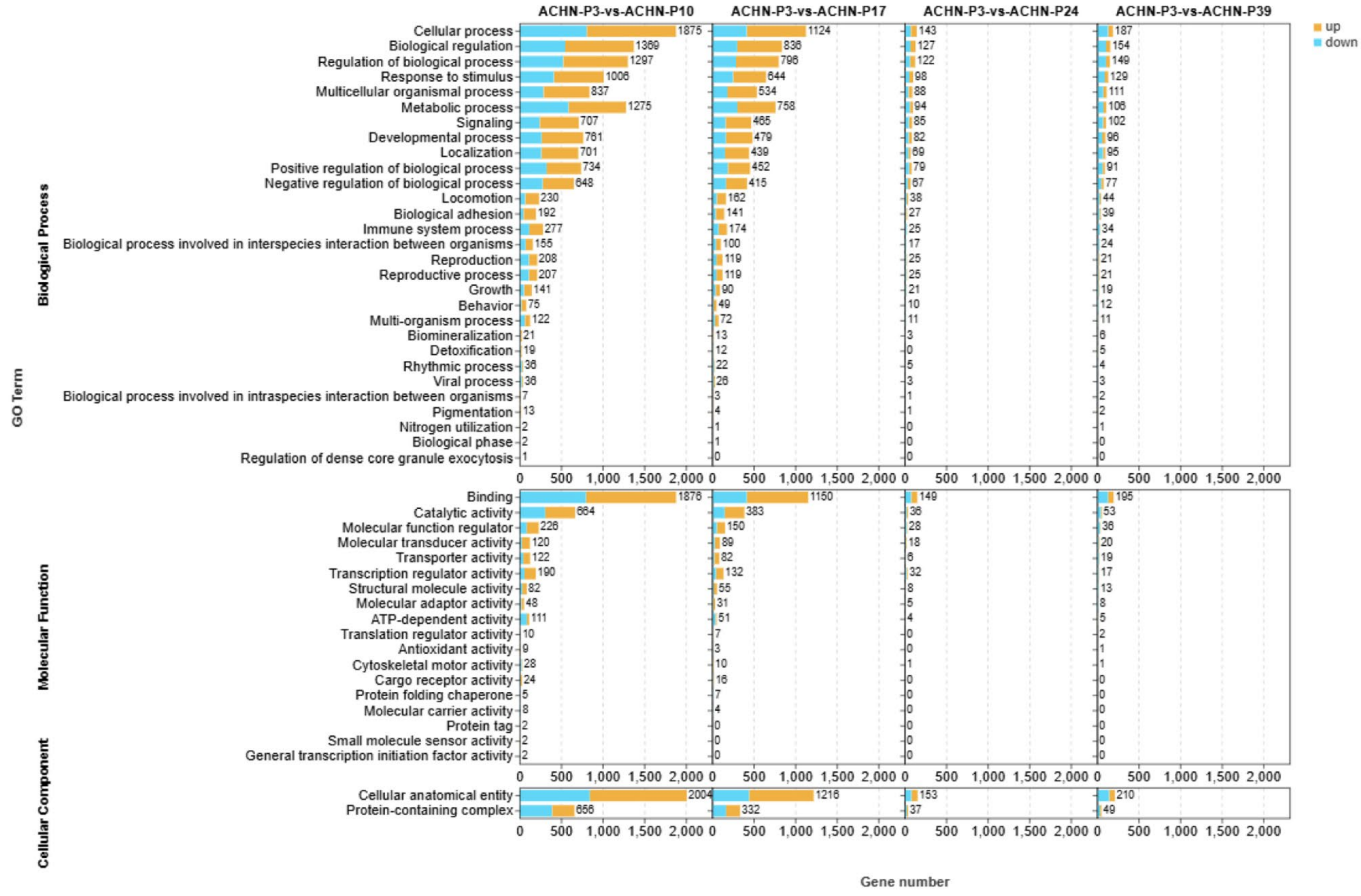
GO enrichment analysis reveals functional changes of DEGs in ACHN cells at different passage stages. GO enrichment results of DEGs identified in ACHN cells at different passage comparisons (P3 vs. P10, P17, P24, P39) across three major categories: Biological Process, Molecular Function, and Cellular Component. Orange bars represent enrichment terms for up-regulated genes, while blue bars indicate terms for down-regulated genes. GO terms are sorted by gene count, showing a decreasing trend in both the number of enriched terms and associated genes with increasing passages.

**Fig. 5:**
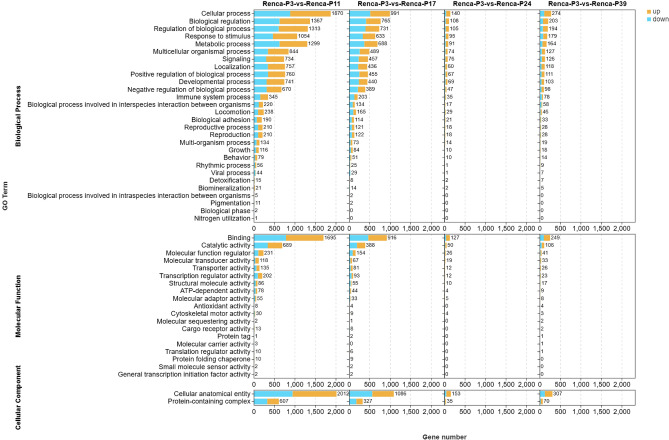
GO enrichment analysis reveals functional changes of DEGs in Renca cells at different passage stages. GO enrichment results of DEGs identified in Renca cells at different passage comparisons (P3 vs. P11, P17, P24, P39) across three major categories: Biological Process, Molecular Function, and Cellular Component. Orange bars represent enrichment terms for up-regulated genes, while blue bars indicate terms for down-regulated genes. GO terms are sorted by gene count, showing a decreasing trend in both the number of enriched terms and associated genes with increasing passages.

To investigate the potential biological functions of DEGs during continuous passaging, we performed Gene Ontology (GO) enrichment analysis on DEGs identified at different passage stages in both ACHN (Fig. 4) and Renca cells (Fig. 5). The results revealed that in both cell lines, DEGs between low and intermediate passages (P3 vs. P10/P11) were primarily enriched in fundamental biological processes such as cell cycle regulation, biological regulation, metabolic processes, and response to stimulus. This suggests that cells at intermediate passages exhibit heightened transcriptional activity and regulatory sensitivity. As passage number increased, the number of significantly enriched GO terms gradually decreased. Particularly in comparisons between P3 and later passages (P24, P39), only a limited number of functional categories remained enriched, indicating a substantial reduction in both the quantity and functional diversity of DEGs. This trend aligns with the observed decrease in DEG numbers described earlier. At the molecular function level, enriched terms were predominantly associated with binding, catalytic activity, and transporter activity. Cellular component analysis revealed enrichment in cellular anatomical entities and protein-containing complexes (Supplementary Material 2).

These findings suggest that continuous passaging may lead to a gradual loss of functional diversity and responsiveness, reflecting a stabilization trend in the transcriptional profiles of long-term cultured cells.

### KEGG enrichment analysis of DEGs during cell passaging

**Fig. 6:**
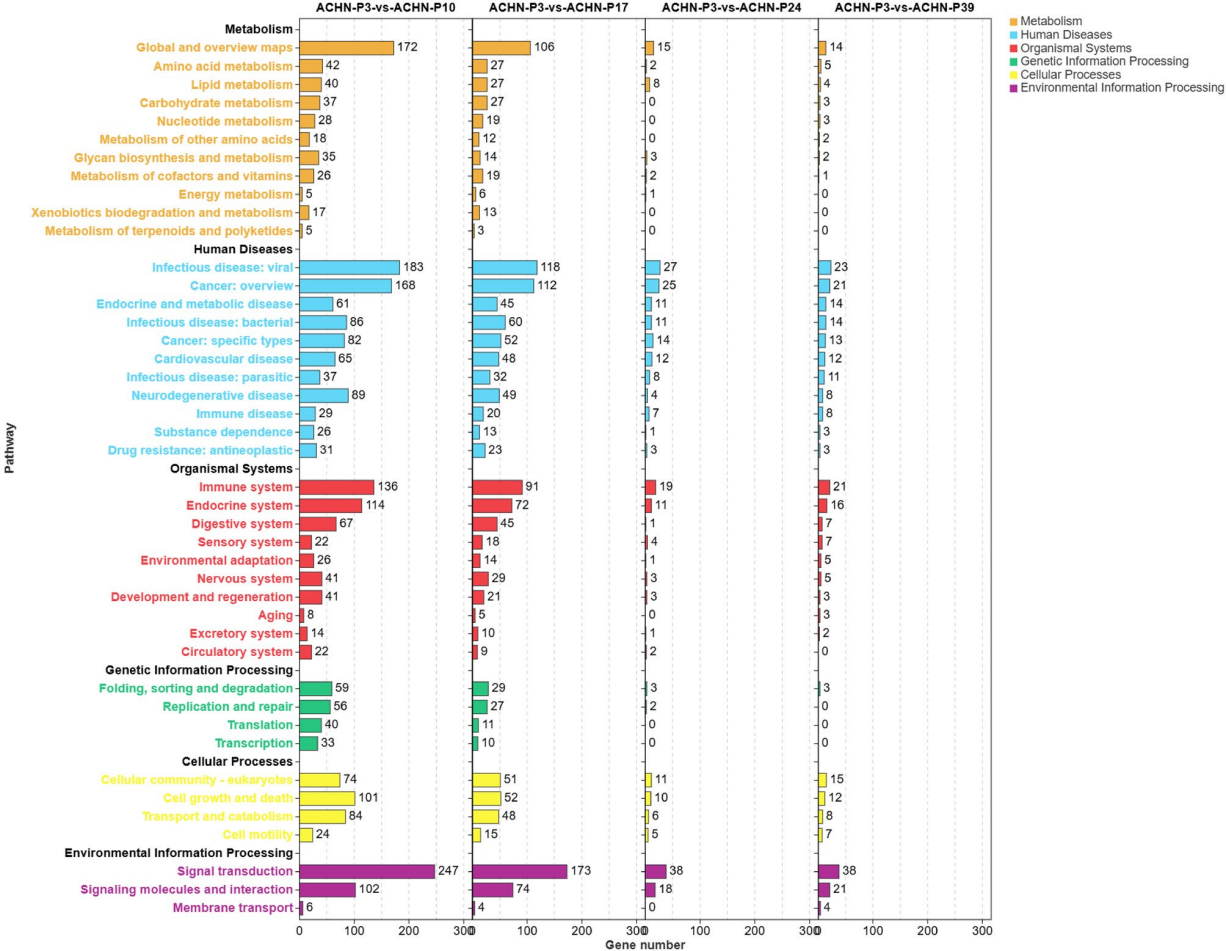
KEGG pathway enrichment analysis of DEGs in ACHN cells at different passage stages^20^. DEGs from comparisons between ACHN-P3 and P10/P17/P24/P39 passages were subjected to KEGG pathway enrichment analysis. Pathways are color-coded by functional categories: metabolism (orange), human diseases (blue), organismal systems (red), genetic information processing (green), cellular processes (yellow), and environmental information processing (purple). The x-axis indicates the number of enriched DEGs in each pathway.

**Fig. 7:**
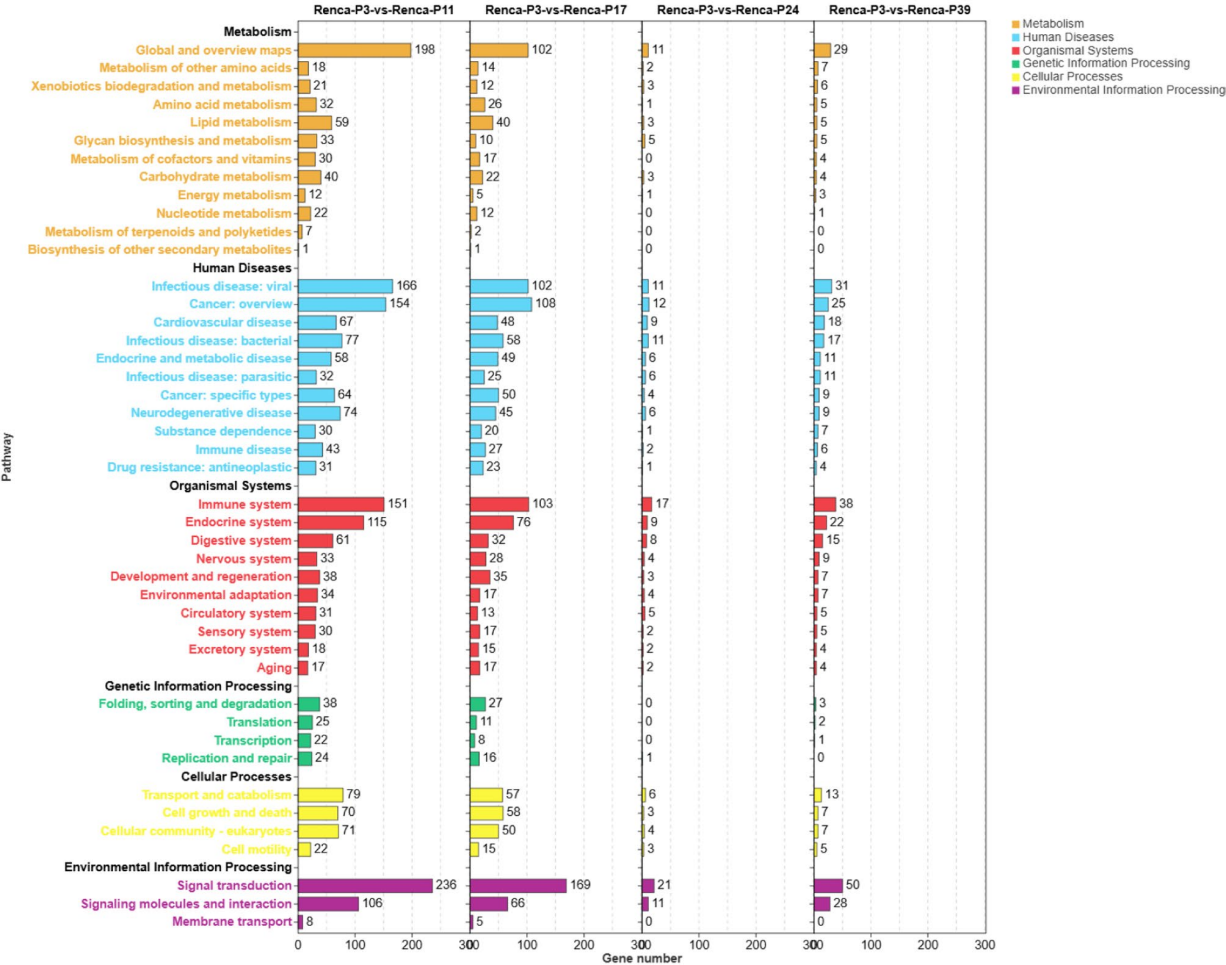
KEGG pathway enrichment analysis of DEGs in Renca cells at different passage stages^20^. DEGs from comparisons between Renca-P3 and P11/P17/P24/P39 passages were subjected to KEGG pathway enrichment analysis. Pathways are color-coded by functional categories: metabolism (orange), human diseases (blue), organismal systems (red), genetic information processing (green), cellular processes (yellow), and environmental information processing (purple). The x-axis indicates the number of enriched genes in each pathway.

To investigate the dynamic molecular responses of renal cancer cells during passaging, we performed KEGG pathway enrichment analysis on DEGs from ACHN and Renca cell lines at multiple time points. In ACHN cells (Fig. 6), DEGs between low (P3) and intermediate (P10) passages were significantly enriched in multiple key pathways, including signal transduction (247 genes), infectious diseases (183 genes), global metabolism and overview pathways (172 genes), and immune system (136 genes). Additional enrichment was observed in endocrine metabolic diseases, cancer-related pathways, and transcriptional regulation. As passage number increased (P17 to P39), both the number of enriched pathways and DEG counts markedly decreased, indicating a diminished transcriptional response in ACHN cells. However, environmental information processing pathways (e.g., signal transduction and membrane transport) maintained certain enrichment levels. Renca cells (Fig. 7) showed a consistent trend, with broad pathway activation observed when comparing low (P3) and intermediate (P11) passages. Significant enrichment was particularly notable in signal transduction (236 genes), infectious diseases (196 genes), global metabolism (198 genes), and immune system (151 genes) (Supplementary Material 2).

In summary, both tumor cell lines exhibited similar patterns of transcriptomic changes during passaging. Pathways related to signal transduction, immune response, and metabolic regulation were widely involved in cellular responses at intermediate passages, suggesting that intermediate passage stages may represent the optimal window for detecting drug intervention effects.

## Discussion

Cancer cell lines serve as fundamental research platforms, generating data that underpins numerous studies and increasingly fuels artificial intelligence and deep learning applications. However, the reproducibility of experimental results has long been a critical challenge hindering scientific progress^1^. Identifying factors contributing to irreproducibility is therefore essential for advancing research.

The stability of cell-based experimental outcomes is significantly influenced by various extrinsic factors, with cross-contamination, mycoplasma infection, and fetal bovine serum (FBS) variability being the most prominent concerns. Cross-contamination remains prevalent in cell culture practices; a study of 360 cell lines revealed that HeLa cells were the most common contaminant (29%), while cross-species contamination accounted for 9%^2^. To address this, Yu et al. proposed establishing standardized authentication methods based on STR and SNP profiling, integrated with cell line databases to enhance experimental reliability ^3^. Mycoplasma contamination represents another major interfering factor. It non-specifically suppresses mRNA expression of IL-1α, IL-1β, and actin in certain cell lines (e.g., A431)^4^, activates the TLR2/NF-κB pathway through immunogenic lipoproteins^10^, and alters the expression profiles of hundreds of genes ^5^. More severely, it disrupts dendritic cell differentiation, characterized by downregulated DC-SIGN and upregulated CD123—effects reversible upon contamination clearance^6^. Mycoplasma contamination reportedly costs the scientific community millions annually^7^. Furthermore, FBS source variability significantly impacts reproducibility. Different FBS brands markedly alter baseline IL-8 expression in epithelial cells^9^, while its harvesting methods raise ethical concerns^8^. These factors collectively constitute major extrinsic sources of variability, necessitating standardized detection protocols, rigorous quality control, and development of alternative materials to systematically address these challenges.

Beyond extrinsic environmental factors, the stability of cell-based results is profoundly constrained by intrinsic biological characteristics. Studies demonstrate that long-term passaging can alter cell morphology, proliferation capacity, and functional properties. For instance, RAW 264.7 macrophage cells maintain stable phenotypes and functions within passages 10–30, but reliability declines beyond passage 30^11^. Equine bone marrow mesenchymal stem cells (BM-MSCs) retain stable immunomodulatory functions from P3 to P9, but significantly increase pro-inflammatory cytokine secretion at P9^12^. Such passage-related changes often exhibit nonlinear patterns, where low- and mid-passage cells may share closer characteristics than with extremely high passages^13^. Metabolomic analyses reveal that passage number significantly influences cellular metabolic networks. In RAW 264.7 cells, low-passage (P9) cells digested with trypsin show higher metabolite signal intensity, with glycolysis and TCA cycle pathways markedly affected by passaging, while glycerophospholipid metabolism is primarily influenced by cell collection methods^14^. Similarly, dental pulp stem cells (DPSCs) gradually lose normal morphology with passaging, and late-passage cells exhibit reversed responses to drugs such as alendronate^15^. Notably, passaging effects are particularly pronounced in stem cell properties. In non-small cell lung cancer (NSCLC) cells, late-passage side population (SP) cells show significantly diminished cancer stem cell characteristics^16^. These findings emphasize the need for strict standardization of passage ranges in experimental design. For example, Hanwoo muscle satellite cells maintain optimal differentiation potential within P1-P10^17^, while mouse embryonic fibroblast (MEF) culture requires comprehensive consideration of strain, gestational age, and passage number^18^. Additionally, Liu et al. demonstrated that genotypic variability induces substantial proteotypic and phenotypic variations in identical cell lines cultured across different laboratories, representing another critical factor contributing to irreproducibility^19^.

This study systematically analyzed transcriptomic dynamics in two tumor cell lines (ACHN and Renca) across passages (P3 to P39) using RNA-seq. We identified a nonlinear pattern of gene expression, defined as significant intermediate fluctuations coupled with relative stability at both ends. Functional enrichment analysis revealed that differentially expressed genes were primarily associated with active biological processes such as cell cycle regulation, metabolism, and stress response. KEGG analysis further uncovered disparities in signaling transduction, immune response, and metabolic pathways between passages, providing novel insights into the intrinsic biological mechanisms underlying experimental irreproducibility.

In conclusion, to advance scientific research, we recommend strict control of cell passage numbers and explicit reporting of this information in publications. These measures represent low-cost yet effective strategies to significantly enhance the reproducibility of cell-based experiments.

## Limitations

This study provides the first systematic characterization of nonlinear transcriptomic dynamics during serial cell passaging, offering important insights into cellular heterogeneity in experimental systems. Several limitations should be acknowledged. First, the generalizability of our findings is constrained by the analysis of only two renal carcinoma cell lines (human ACHN and mouse Renca), which may not fully represent the behavior of other cell types, particularly primary cells or cancer models from other tissues. Second, the statistical power of our study is limited by the use of two biological replicates per passage point, although the high consistency between replicates supports the robustness of the observed trends. Future confirmatory studies would benefit from larger sample sizes to better account for biological variability.

Methodologically, our study focuses primarily on transcriptomic profiling, leaving the underlying molecular mechanisms—such as epigenetic regulation—unexplored. All experiments were conducted using DMEM medium, and the potential influence of different culture media components on passage effects requires further investigation. Additionally, RNA-seq data alone cannot comprehensively assess genetic drift that may occur during long-term culture. Finally, while we identified significant gene expression changes, establishing direct links between these molecular alterations and functional experimental outcomes (e.g., drug responses or phenotypic changes) remains an important direction for future research.

These limitations highlight several productive avenues for further investigation, including validation across diverse cell models and culture conditions, elucidation of molecular mechanisms, and integration of functional assessments with transcriptomic profiling.

## Supplementary Information

Supplementary Information 1.

Supplementary Information 2.

## Data Availability

The datasets generated and analyzed during the current study are available in the Gene Expression Omnibus (GEO) repository, https://www.ncbi.nlm.nih.gov/geo/query/acc.cgi?acc=GSE308793 and accession number GSE308793.
