# Supplementary figures and images for: Cell passage number drives transcriptomic drift as an overlooked factor in experimental reproducibility
Source: Sci Rep. 2025 Nov 21;15:44984. doi: 10.1038/s41598-025-29424-1 (PMC12748706; doi:10.1038/s41598-025-29424-1)

## Slide 1
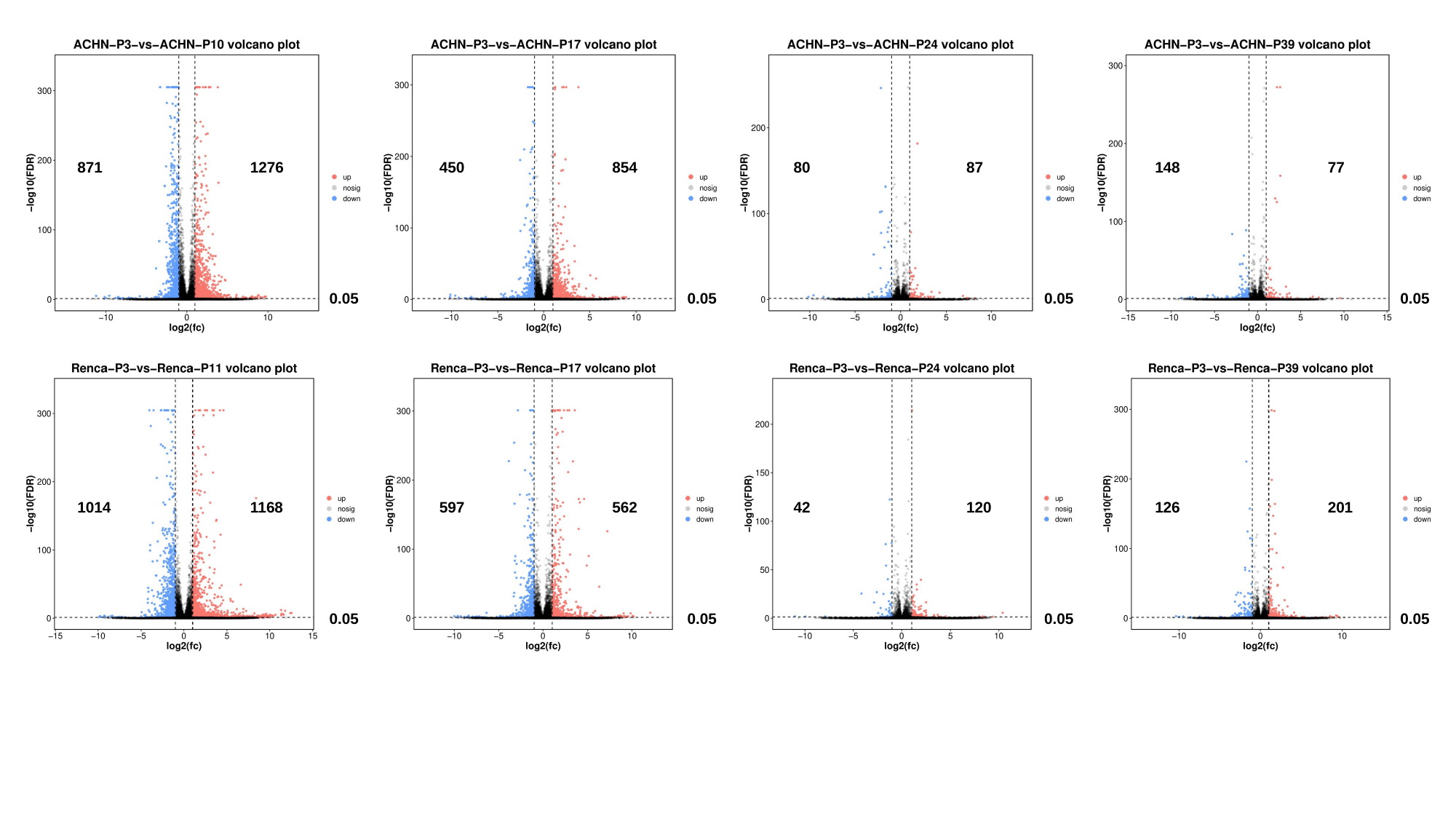

871
1276
450
854
80
87
148
77
0.05
0.05
0.05
0.05
1014
1168
597
562
42
120
126
201
0.05
0.05
0.05
0.05

Supplement: Supplementary file 1 — Supplementary Information 1. [file 41598_2025_29424_MOESM1_ESM.pptx]
